# Skin and Disease in Early Modern Medicine: Jan Jessen's *De cute, et cutaneis affectibus* (1601)

**DOI:** 10.1353/bhm.2020.0034

**Published:** 2020

**Authors:** Hannah Murphy

**Keywords:** skin, disease, Jan Jessen, Holy Roman Empire, surgery, French pox, vernacular print, technique

## Abstract

This article examines skin and disease in early modern medicine through the writings of the little-known Bohemian physician Jan Jessen (1566–1621). In 1601, Jessen published *De cute, et cutaneis affectibus*, a set of twenty-one theses dedicated to the question of whether skin disease existed. In considering Jessen and his relationship to a broader world of writing, this article makes three arguments. First, it suggests that, contrary to existing historiography, the question of skin disease was a common sixteenth-century concern. Second, it posits a professional channel for this concern, which arose from surgery and disease, rather than from anatomy and physiology. Finally, rather than positioning Jessen at the forefront of discovery, I suggest his text functions as a representative case study. It allows us to see material change in medicine within a stable Galenic framework.

In 1600, a disputation took place at the University of Wittenberg on the subject of skin disease. The theses were put forward by Jan Jessen, rector of the university, professor of anatomy, surgery, and botany, and author of a series of texts on anatomy, surgery, and natural philosophy. They were [Other P-179] defended by Johann Cögeler.^1^ The result of the disputation is unknown, but a year later the question was still on Jessen's mind, and he had his theses published by the university printer. The resulting work, *De cute, et cutaneis affectibus* (On skin and the conditions of skin), appeared in Wittenberg in 1601 (Figure 1).^2^ Nearing the end of his tract, Jessen summed up his chief provocation: "The true controversy is whether to call [such] faults diseases of the skin."^3^ It was a question to which his theses answered an unequivocal yes.

The question of skin disease was a key concern of the sixteenth century, and it mattered because it was not Galenic, a fact that Jessen addressed directly. As historians of early modern medicine will know, there was no clear-cut definition of disease in the sixteenth century.^4^ In humoral medicine, ill health resulted first and foremost from an imbalance of humors and complexions.^5^ The question of whether and what part of the body could be affected by disease was thus problematic. As per Galen, for early moderns, disease of a body part meant it was acting "contrary to nature," or against its own purpose.^6^ When it came to skin, this explanation was even more problematic because according to classical medicine skin had no primary purpose. Neither Aristotle nor Galen provided a real definition of skin, reverting to metaphors or euphemisms in attempts to explain its role. In the first published treatise on skin, which appeared in 1572, the Italian Girolamo Mercuriale (known in Germany more commonly[Other P-180]

**Figure 1:**
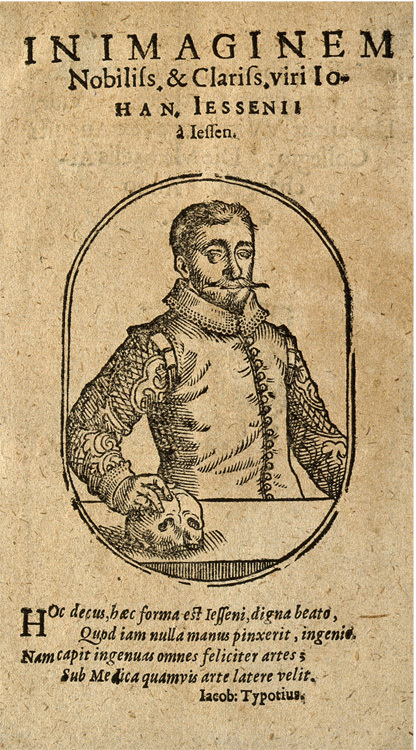
Jan Jessen (1566–1621), half length, resting his right hand on a skull.

[Other P-181] by the Latinate Hieronymus Mercurialis) described skin in the tradition established by Plato, as a "fisherman's net."^7^ According to Mercurialis the porosity of skin functioned primarily to facilitate emissions of waste or to protect the flesh from absorbing harmful matter in the air. In these theoretical terms skin could *display* disease, but it could not itself *be* diseased. Alterations to the skin—boils, ulcers, rashes, and so forth—were the product of harmful or morbid matter within the body, pushing its way to the surface. They were therefore signs of disease elsewhere, but they were not signs that skin was acting contrary to its nature or purpose. On the basis of this at least one historian of medicine has concluded that in early modern medicine "a perception of 'skin diseases' . . . existed only to a limited extent."^8^

But while classical Galenic theory may not have clearly delineated a category of skin disease, for early moderns the secondary purposes of skin, that is, its role in supporting the vital functions of the body, rendered the status of skin disease more ambivalent. As Andrew Wear has clearly shown, in early modern practice the modern distinction between symptom and disease played little role.^9^ As a surface for displaying disease, skin played an increasingly important role in diagnosis, prognosis, and treatment throughout the sixteenth century. In literature on pox and plague, in vernacular works of surgery, in pharmaceutical texts and anatomical treatises, not to mention in the social circumstances of everyday life, patients insisted that skin was the surface through which their own knowledge of disease was made evident. Practitioners as well as patients routinely used skin as a surface for diagnosis, but also for prognosis and for treatment. The concepts and categories they used did not fit neatly into modern ideas of body parts, organs, or pathological signs and symptoms, but neither did they adhere straightforwardly to Galen.

This article examines the emergence of skin as a medical subject over the course of the sixteenth century, mostly (but not exclusively) in the Holy Roman Empire. As a case study for the making of medical knowledge, the emergence of skin disease presents a narrative removed from moments of discovery. Current work on skin in early modern physiology has positioned its emergence within the narrative of anatomical "discovery," facilitated most importantly by the microscope.^10^ In fact, I suggest the opposite: medical knowledge of skin arose from concern with surgery [Other P-182] and disease, rather than from anatomy and physiology. This article makes three related arguments. First, and most fundamentally, it argues that the existence of skin disease was in fact a sixteenth-century concern. Far from appearing as an outlier, Jessen's text was keen to emphasize a wide scholarly consensus about the importance of skin. In this, Jessen was responding to two major developments of the sixteenth century. One was the conceptualization of disease itself. The other was the emergence of skin as a site of medical practice. The first part of this article examines these in tandem, first in the context of Galenic medical writing, and then in vernacular literature. As we shall see, widespread acceptance of skin disease accorded far more nearly to the layperson's conception of what could be read on the body than to the Galenic definition of what was contrary to nature and purpose. There is thus a relationship between disease concepts and skin. As one changed, so did the other, and as one was contested, so was the other.

The second point this article makes addresses the practical concerns underlying writing about skin. It argues that as a learned physician, interested in surgery and anatomy, and writing for the purposes of attracting patronage, Jessen represents a professional channel for this concern with skin and disease, one that existed within the scope and sphere of learned Galenic medicine. Skin arose primarily from the treatment of disease, to be co-opted by physicians with an interest in the overlapping fields of surgery and anatomy. By positioning these together, Jessen opens a window onto the role of practice in Galenic medicine and the way in which scholarly writers incorporated it into the canon of medical learning.

The third point exists at the intersection of the previous two and involves the way in which learned medicine encountered and changed in the face of commonplace practice. In arguing for the recognition of skin disease as a category, Jessen was on the one hand performing the core concern of sixteenth-century learned medicine: the reconciliation of practice with Galenic medical theory. But Jessen did more than simply attempt to reconcile theory and practice. He also reoriented the analytical framework of medicine toward practice. The grounds for Jessen's argument were not simply that medical practitioners encountered skin disease and thus medical theory should accommodate it as a category. He argued, more specifically, that the perception of skin disease was ubiquitous and thus medical theory should expand to encompass it. For Jessen to arrange the Galenic categories of disease around the precondition of commonplace, practical concerns provides an insight into not only the way in which early modern physicians responded to practical shifts in the study of medicine but also how they thought about those shifts. His argument for learned recognition [Other P-183] of a commonplace matter of practice presents a powerful reminder to historians of medicine that before medical paradigms arose in print or were consciously articulated as developments, they often stemmed from and responded to long-standing categories of practice.

**Figure 2:**
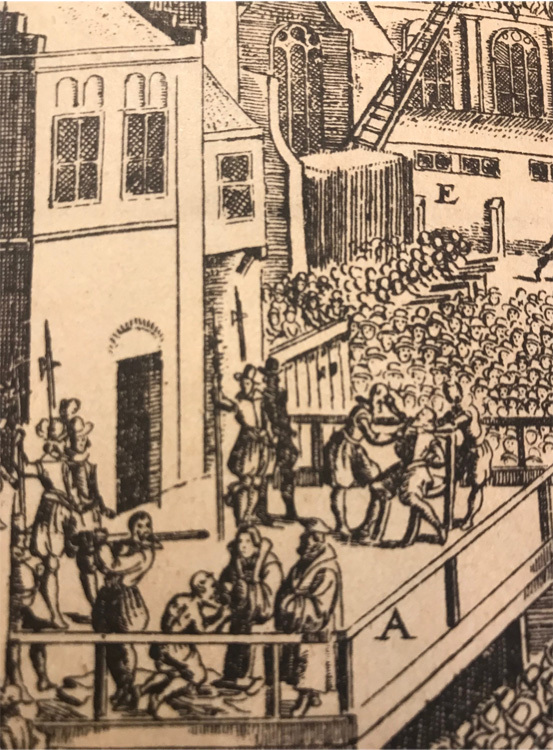
Detail, *Eigentliche Abbildung dess Process der Pragerischen Execution* (1621).

[Other P-184]

## Jan Jessen: Practice and Learning in Sixteenth-Century Galenic Medicine

In 1621, Jan Jessen was executed along with twenty-six other Protestant nobles, caught up in the political intrigues around the Thirty Years' War (Figure 2).^11^ For this, he remains an important figure in Czech memory, although he is little known in the English-speaking world.^12^ At the turn of the seventeenth century, Jessen was representative of a larger circle of physicians, medical practitioners, and natural philosophers at the Bohemian center of the Holy Roman Empire, whose professional ambitions oriented around the imperial court but who contributed in broad terms toward medical developments and scholarly networks across the continent. More so than Italy, where learned medicine was tied to well-established institutions such as universities, guilds, or *collegia medica*, medicine in sixteenth-century Germany was still in the process of organizing, and partly because of this, it involved a complex intermingling of scholarship and empirical practice at all professional levels.^13^ Jessen moved with ease between academic and nonacademic contexts, seeking patronage, often by means of published texts. While none of his texts are remembered as particularly groundbreaking, they are characteristic in expressing how learned and practicing physicians across the Holy Roman Empire contributed to the making of medical knowledge in a European context.[Other P-185]

Jessen's biography is suggestive of this emerging category of learned practitioners, who I have recently suggested constituted a "new order" of medicine.^14^ Born in Breslau in 1566, like many of his Bohemian peers, Jessen studied in Wittenberg and then in Italy, marrying the Melanchthonian traditions of reformed, Aristotelian natural philosophy to the incipient schools of anatomy, botany, and empirical observation that characterized Italian medical education.^15^ More specifically, Jessen went to Padua for his medical degree, exposing him to a complex medical context, where, as much recent scholarship has shown, competing ideas about anatomy, surgery, and medicine were being worked out in practical and intellectual ways.^16^

When Jessen arrived in the 1580s, Padua enjoyed an unparalleled reputation as a center of anatomical expertise and experimentation. However, as Cynthia Klestinec has shown, the nature of that expertise and experimentation was under debate.^17^ As the former home of Gabriele Zerbi and Andreas Vesalius, Padua had been at the forefront of Italian teaching in anatomy as well as in the staging of public dissections. By the time Jessen arrived in Padua, Girolamo Fabrici d'Aquapendente possessed the ordinary chair in anatomy, coming under frequent criticism for his reluctance to hold public anatomies.^18^ But while the status of anatomy was in question, Padua was also home to a cluster of physicians and surgeons increasingly linking medicine with surgery and new practices of the body.^19^ These included Giovanni Thomaso Minadoi and Gaspare Tagliacozzi, Girolamo Capivacci, Emilio Campolongo, Albertino Bottoni, Marco degli Oddi and [Other P-186] Paolo Galeotto, and, most notably for skin, Girolamo Mercuriale.^20^ The changing status of anatomy and the emerging focus within this group on surgical approaches to disease help clarify the distinction between anatomy and disease-driven physiology as an impulse to thinking about skin.

This is especially relevant given current scholarship on the symbolic representation of skin. In recent years the field of "skin studies" has brought together cultural and psychological ideas about skin and its historical representation to examine notions of the individual. Scholars such as Claudia Benthien and Steven Connor have drawn attention to the prominence of skin in early modern visual imagery, literary tropes, and ritual practices from relics and the statues of saints to branding and flaying.^21^ A key feature of such work is the detection of a new interest in skin in the sixteenth century. Medicine might seem like an obvious source to support such assertions. Art historians including Daniela Bohde and Mechthild Fend have shown how artistic preoccupation with skin was intimately connected to changing anatomical knowledge.^22^ And yet while a rich body of work now exists on the symbolic representation of skin, there has been little to no work on how medical practices around skin changed in this period or how new medical conceptions of skin emerged.^23^

Changes in medical understanding of the importance of skin did not come from anatomy, where neither skin nor functional understandings of skin was a focus on inquiry. In part, this is because the status of skin within medicine, as the introduction to this article has already mentioned, [Other P-187] was unclear. Under Aristotle's characterization, to be part of the body was to partake of its life. For Galen, this was more direct: having a clear bodily purpose. Both Galen and Aristotle distinguished between *cuticula* (*epidermis*) and *cutis* (*dermis*). Cuticula, the top-most dried-out flesh of the body, had no clear bodily purpose. Cutis, the spermatic layer beneath, was more ambiguous. Despite not performing a specific function, cutis performed secondary functions: it protected the body from external injuries, kept its natural inner warmness, helped excretion, and gave the body its beauty.^24^ When he performed his famous anatomy, Vesalius referenced the established division between cuticula and cutis (as well as the *panniculus*, a fatty membrane that separated skin from "flesh"), but rather than explaining or interrogating the function of skin, Vesalius avoided explanation.^25^ When it came to the physiology of skin, Vesalius reverted to analogy, comparing it to a house, with beams and paneling, configured in terms of proportion and function.^26^ (Ironically, in the treatises on architecture from which Vesalius borrowed, authors such as Alberti and Vitruvius borrowed the metaphor of skin to think through the appearance of houses.)^27^ Despite the apparent focus on skin inherent in anatomy, skin was not an anatomical subject. The visual motif of the écorché encapsulates this perfectly, presenting the skin removed intact to enable dissection of what lies beneath, rather than itself a subject of dissection and interrogation.

Instead, where interest in the function of skin and its relation to the human body could be found was in treatises on disease. Sitting at the exterior of the body, but not clearly a boundary, skin formed part of the physicians' dilemma—how to diagnose the interior of the body via exterior signs.^28^ The nature of skin thus spoke to the emblematic, analogical nature of medicine as well as to the traditional elements of diagnosis, [Other P-188] prognosis, and treatment in which medical thinking resulted. This was already evident in the medieval manuscript tradition, where skin was more typically elaborated on in curative texts by Celsus, Avicenna, and Isidore of Seville, among others. Book II of Celsus's *De Medicina* (On Medicine) considered diseases, and within this category Celsus took care to distinguish diseases of the scalp from diseases of the face, and included skin blemishes as diseases. Book III included influential consideration of elephantiasis, and Celsus also featured skin as a descriptor in a whole variety of other diseases, including scrofula and ulcers. Isidore of Seville (ca. 560–636) grouped skin diseases together, considering a category of "diseases that appear on the surface of the body," which included alopecia, parotids, lentigo, serpigo, impetigo, prurigo, nytcalopia, warts, scabies and lepra, elefantiasis, jaundice, cancer, ulcers, pustules, imples, fistuals, and scars.^29^ Book IV of Avicenna's Canon considered diseases that affected the "whole body." This included smallpox (*de variolis*), measles (*de morbillo*), leprosy, and a huge number of inflammatory conditions, including "Persian Fire" or anthrax (*de pruna et igni persico*), most of which he stated took place on the skin, but none of which he considered to be skin diseases per se, as well as diseases of the hair and scalp.^30^ As we shall see below, distinguishing between specific skin-related areas, such as the scalp, while including such afflictions under the category of the whole body was a feature that would be elaborated in sixteenth-century texts.

None of these authors defined a disease *of* the skin, but what they do make clear is how central skin was to diagnosis, prognosis, and treatment of disease. Possibly aided by the first printed editions of Celsus toward the end of the fifteenth century, the sixteenth century saw novel developments in the print history of skin.^31^ The most obvious was the publication of Hieronymus Mercurialis's *De Morbo Cutaneis* (On Diseases of Skin), the 1572 treatise often cited as the "earliest dermatological treatise."^32^ In the introduction to his text, Mercurialis addressed the distinction between diseases of the "whole" skin and diseases of the scalp and head. He progressed [Other P-189] through these in two books, following roughly the head-to-toe model, describing the disease in question as well as his attempts to treat it.

Despite the title of his text, Mercurialis explicitly refrained from adopting a new definition of disease or arguing for a discrete category of skin disease. He also reiterated Galen's central conclusion that the skin does not have common or primary functions within the body. And yet, using Galenic categories, Mercurialis insinuated a more refined taxonomy of skin diseases within existing theory. "It seems to me," he wrote, "that diseases of the entire skin are of three types: those of color, of roughness or smoothness, and of bulk."^33^ Diseases in these types did not affect the consequential actions of skin, and so did not count as skin diseases per se. Instead Mercurialis used the category of *turpitudines* (disfigurements) to describe them, referencing Plato's concept of disharmony. As a disharmony, turpitudines existed as the opposite of pulchritude. Pulchritude was health; turpitudines were thus ill-health or disease.^34^ While Mercurialis never explicitly broke with Galen, he marks the entrance of this category into the printed domain as a subject. And he appears as an excellent example (as Nancy Siraisi has remarked in other respects) of the characteristic tightrope walk performed by physicians, modifying Galen in practice while simultaneously shoring up Galenic medicine as a category of learning.^35^

Mercurialis's writing also brings to the foreground a very simple point—that as well as being expected to read signs on skin and diagnose complex diseases, physicians were frequently confronted by ailments, blemishes, and cosmetic disfigurements and that they were expected to treat these as if they were diseases (regardless of whether theory made room for such a fact). In this, Mercurialis foreshadows Jan Jessen, suggesting that his treatment of skin and the categorization of disease was in keeping with how many learned physicians encountered the expectations of patients. It certainly fit neatly into his broader publishing habits. In general, Mercurialis's published texts tended to focus on elements of common practice, which he then attempted to elevate to the status of learned medicine. For example, between 1552 and 1601, Mercurialis wrote three texts on children and women as well as important texts on pharmacy, poisons, and plague and the first tract on orthopedics, "The Art of Gymnastics."^36^ In addition, as well as *De morbo cutaneis*, Mercurialis [Other P-190] also wrote *De decoratione* (On Adornment), a text on cosmetics, which is of no less importance to thinking about skin.^37^ Not only did *De decoratione* include significant detail on skin ailments on the face and hands, it also expounded in more detail the grounds for considering skin's importance to the body in aesthetic terms, as a vital component in the maintenance of the body's harmony.^38^ Read as practical texts, *De decoratione* and *De morbo cutaneis* present a professional trajectory, one in which it is clear that Mercurialis encountered skin ailments and then went to great lengths to "claim" them within the physicians' domain.

In her important article "Boils, Bumps and Wheals," Olivia Weisser showed how the expansive category of "bumps" formed part of the collaborative experience of reading the body in the seventeenth century, one that linked patient and practitioner.^39^ Here, I suggest that a recognition of this collaborative experience of reading the body informed many of the assumptions behind sixteenth-century learned medical texts, which then addressed them in a context shaped by professional ambitions. This commonplace, ubiquitous focus on skin was something that would be particularly key for Jessen. But it also raises a question mark regarding the world of skin to which physicians were reacting—a world of vernacular writing and artisanal practice in which skin functioned as a frontline in different ways.

## Skin and Disease in Early Modern Germany

Because of Mercurialis, the early modern medical treatment of skin has been framed in traditional terms, when it is recognized at all, as the learned by-product of a university context or more recently as arising from the Renaissance meeting of medical commentaries on Galen with new epistemes of observation.^40^ But it should already be obvious that the learned presentation of such ideas did not happen in a vacuum but rather [Other P-191] built on and responded to changes in the underlying practices of medicine. These included medical responses to disease and treatment, but also new methods of diagnosis and prognosis. In Germany more so than in Italy, these changes were first articulated in vernacular books rather than in the context of the university.

A critical feature of the social context for medicine in Germany was the vibrant market for print, which was continually expanding.^41^ German publishing produced a rich well of vernacular medical literature, written by barber-surgeons and lay practitioners as well as by learned physicians.^42^ Such books included tracts on exceptional diseases such as plague or pox, tracts on specific diseases such as gout, compendia of diseases (often following the "head-to-toe" model), surgical and pharmacological texts offering cures to disease, and eventually the *observationes*—a new genre of texts that offered clinical case histories on rare and new diseases and important cures.^43^ In all of these, it appears evident that closer scrutiny of skin grew out of concern for its treatment; and the physiological developments that did indeed take place at this time came from the question not of how the body worked (anatomy, in its proper sense) but of how it might be healed (medicine and, increasingly, surgery).

Much has been written about this rich tradition of vernacular print and German medicine.^44^ The kind of anarchic creativity it could facilitate can be glimpsed in the innovations at work in well-known texts, such as *Feldbuch der Wundartzney* by Hans von Gersdorff, a field book of barber-surgery that appeared in Strasbourg in 1517.^45^ Based on the *Chirugia magna* by Guy de Chauliac, *Feldbuch der Wundartzney* contained, among other important things, an interesting innovation on the subject of skin.^46^ [Other P-192] The first paragraph described it: "So it is therefore to look at the skin, as when you are caring first and foremost for the outside of the bark of a tree. And it is the cover of the body, in which the senses and blood sit together."^47^ The original text by de Chauliac stopped short of this, stating merely that skin was the cover of the body, or, as the 1525 English translation describes, "It is to begynne atte be skyn, for it renness first in makynge of the anothomye. The skyn forsope is to be couerynge of ye body, iwoven of thredes of pe synowes, of pe veynes."^48^ The analogy drawn by Von Gersdorff is important because it was an innovation, an imaginative and conceptual neologism. Likening skin to the bark of the tree spoke to the difficulties of definition, but also to the vitality of the artisanal and vernacular tradition of natural philosophy and thinking on the natural world.^49^ Von Gersdorff's analogy of the tree was in keeping with the agricultural tradition established by Columella (whose work was translated into German and printed by the same printer in Strasbourg several years later), and as Paolo Savoia has shown, the concept went on to become an important Renaissance trope, playing into later treatises on skin techniques such as grafting by the Italian Gaspare Tagliacozzi.^50^

Such minor innovations are symptomatic of the kinds of conceptual changes, often fragmentary and frustratingly unsystematic, that early modern vernacular texts can convey. I suggest that, taken together, they show an increasing focus on skin from a triangulation of three literary forces: pox, barber-surgery manuals, and barber-surgery methods. While it may seem like something of an interlude, addressing this is important because it reveals unsystematic developments that gave rise to something more concrete. Nobody ever characterized the French disease as a disease of the skin. And yet just as scholarship on seventeenth- and eighteenth-century syphilis has tracked a parallel between public concern with venereal[Other P-193]

**Figure 3:**
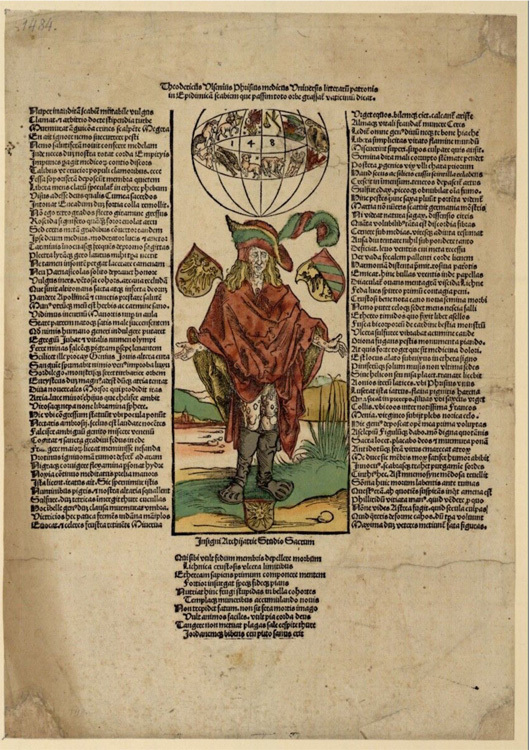
Albrecht Dürer, *The Syphilitic*. Woodcut accompanying the broadsheet by Ulsenius, 1496.

[Other P-194] disease and medical attention to skin, in the sixteenth century attention to pox plotted a trajectory for new attention to skin.^51^ This attention can be seen quantitatively, in terms of the space devoted to it, but also qualitatively, in terms of the emergence of language of (and for) and techniques attempted upon skin.

The main concern of early literature on the French disease was whether pox was a disease and, if so, if it was a "new" disease.^52^ The reason for this was twofold, first because of a lack of textual precedent for the disease and, second, because practical encounters with it proved difficult to diagnose. In 1496, for example, while staying in Nuremberg, the physician Dietrich Ulman (Theodoricus Ulsenius) published an epic poem on the pox.^53^ Like other early commentaries on pox, the narrative of the poem detailed the origin of the disease, but its descriptions clearly focused on skin. The first lines commented on the scabies, and throughout the poem, color, pus, and so forth featured prominently.^54^ The poem was accompanied by a woodcut attributed to Albrecht Dürer, which clearly visualized these signs, providing a visual signpost to the literary emphasis within (see Figure 3). Skin was therefore key to answering the question of whether the pox was a disease.

Historians have shown that the language of pockmarks, pustules, sores, and scabies quickly stabilized, becoming a trope reported by patients as well as recorded by chronicles. "The Bianchina chronicler of Bologna talks of it having 'eaten [away] the nose and half the face,' while Sigismondo dei Conti says that these pustules and ulcers 'gnawed away as far as the marrow.' Matarazzo talks too about the body rotting inside. Indeed one Perugian merchant is supposed to have been so consumed by this disease between the thigh and the torso such that it was possible to see everything he had inside his body and there was such a great hole that it would have been possible to introduce an 'ancrestana' inside."^55^ German patients were[Other P-195]

**Figure 4:**
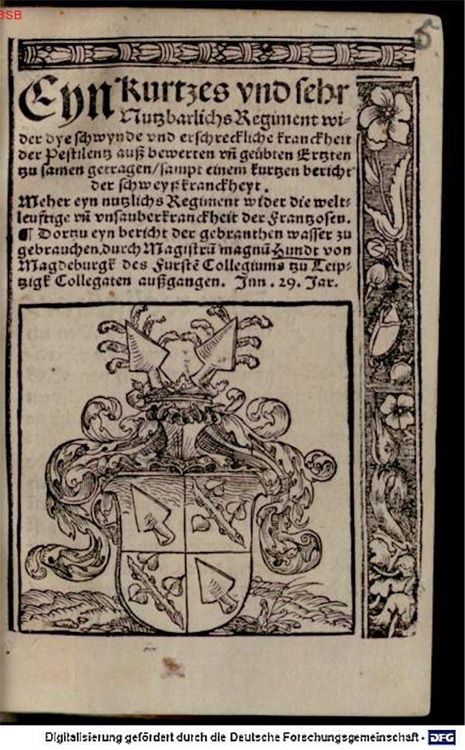
Magnus Hundt, *Eyn Kurtzes und Sehr Nutzbarliches Regiment wider die schwynde und erschreckliche Krankheit der Pestilenz* (1529).

[Other P-196] similarly graphic. Ulrich von Hutton, for example, listed other ailments of the skin with which he believed pox, on account of its common site of occupation, must share sympathy, including "tumours, leprosy, scabia and all kinds of evil scabs and boils with whatever could afflict man's body as well as podagra and many infirmities of the hands and limbs."^56^

Not only were the signs of skin increasingly problematized by this, the matter of skin was increasingly medicalized to think about it. In 1529, the physician Magnus Hundt, better known for his 1503 *Anthropologia*, published *A Useful Regimen Against the Deadly and Terrifying Pestilential French Disease* (Figure 4).^57^ Hundt devoted the first chapter of his regimen to addressing the cause of the disease, which he located in the moral malaise of the day, but more specifically in planetary conjunctions and poisonous air.^58^ Hundt's primary lens for treatment was through the application and management of the six nonnaturals.^59^ Only after that were surgical methods employed. These included bloodletting, providing "relief" from the disease by means of pharmaceutical distillations, including new remedies such as *terra sigillata* and camphor-burning pomanders, and techniques of wound management for the sores or "scabies" caused by the disease.^60^ It was up to medical practitioners to determine the suitability of more complex treatments, and skin color, as key to the body's complexion, played an important role in this. So, for example, bodies that were dry and yellow should not be bled.^61^ Similarly, the pharmaceutical remedies supplied by Hundt were relevant only for particular cases.^62^ Throughout the second half of the text, scabies and skin were both the objects of treatment and the subject of diagnostic attention. Scabies were key to detecting the presence of pox and to assessing and thinking about its progression. Hundt's text contained a variety of surgical interventions to gauge the nature of [Other P-197] sores as well as a variety of methods to treat them. It spoke to the way in which medical practitioners retained a Galenic framework (in the sense of a focus on the origin of disease) while also extending and developing a practice based on intervention, observation, and the appearance of the body. Hundt's attempt to reconcile the pox's pustules in Galenic terms exemplifies this. *A Useful Regimen* explained that the body drove the pox into the skin, which was damaged by the process and so developed the scabies that were called French Pox.^63^

Historians have already noted the impact of the pox on the growing presence of surgical techniques within mainstream medicine.^64^ This large body of literature also had a significant impact on the emergence of the category of skin disease: first, because the symptoms of pox on the skin received significant attention and, second, because in thinking about whether pox was a disease in and of itself, authors were forced to distinguish it from other skin diseases.

In their seminal volume on pox literature in Italy and Spain, Jon Arrizabalaga, John Henderson, and Roger French suggested that the 1520s saw a watershed moment for literature on pox, where writers pivoted from discussing the origins of the disease to discussing its treatment.^65^ This is equally evident in the German literature on the subject, which, as Hundt begins to suggest, pivoted from considering the existence of pox to diagnosing and treating it. It can be seen in the writing of Franz Renner, whose 1557 text, the best-selling *Wundarzneybuch*, justified almost three hundred pages on pox by virtue of its difficult diagnosis.^66^ For many years, Renner wrote, "people suffered and inherited the disease, because we didn't have enough examples. People had gout and we thought it was pox, or pox and we thought it was gout."^67^ Renner's focus was firmly on the difficulty of telling apart pox from other diseases that similarly afflicted the skin. This was more because of the dangers of misdiagnosing the mundanely afflicted, than it was because of missing the specter of pox itself. Renner compared and contrasted the symptoms of other diseases, evident primarily on skin, such as alopecia, scabies, and injured noses (which had [Other P-198] become even as early as the mid-sixteenth century a symptom that signaled pox, but could as easily be caused by age, colds, injuries, and other skin afflictions).^68^ In this manner, Renner explicitly cited the language of case studies. Specifically, he lamented that the difficulty of diagnosing pox lay in the absence of sufficient examples (*Exempel*).^69^ By providing examples, Renner was making a practical contribution to the material treatment of pox.

This change in focus, from the existence of pox to its diagnosis, not only reinforced a focus on skin but multiplied the ways in which skin was considered—not just a surface on which to read signs but as a medical material, to be manipulated, and a means through which to treat patients as well. For example, when attempting to distinguish poxed pustules from more mundane symptoms, Renner concluded that one really had to experiment to test the signs. The disease of pox was in the blood, so he advised lancing the boils to see what material (*Matery*) lay under the skin. He frequently advised remedies that also relied on the methods of barber-surgery, as in chapter 17, when he recommended bathing and smoke for the care of scabies.

Within this broader current of pox writing and pox practice, Renner's text shows how techniques for pox, developed after the specter of epidemics had passed, were then used to reflect on other illnesses, the processes used to detect them, and the cures used to treat them. The use of surgical techniques to manipulate the skin in order to procure a diagnosis speaks to a growing acceptance of surgical knowledge in relation to medicine. Renner was also providing a different kind of utility to skin. Skin was the medium by which such observations were made.

While texts on pox provide a relatively extreme example of disease in which skin played a crucial role, the widespread shift I think they speak to can be glimpsed through other sources as well. One notable example can be seen in the *observationes*, the medical "genre" of writing down cases, which became increasingly popular toward the end of the sixteenth century.^70^ As early as 1516, medical authors began compiling accounts of plague writing. Renner's book preceded by only a decade the publication of the most voluminous of these, the 1566 *De morbo Gallico, omnia quae extant abud* [Other P-199] *omnes medico cuiscunque nationis*, which was put together by Luigi Luisini and quickly appeared in learned collections across the continent.^71^ These early accounts featured as cases in collections of observationes, which featured a similar trend toward distinguishing, by comparison of cases, between skin ailments.^72^

For example, in 1567, Johann Weyer, the Flemish court physician to the Duke of Wuerttemberg, published *Medicarum observationum rararum liber unus*, translated into German under the slightly simpler title *Artznei Buch* (Medicine Book, 1580), which provided clinical descriptions for what the physician described as "new" diseases (*medicorum novarum*).^73^ These included scurvy (*Schurbauch*), pestilence, English sweating sickness, and red murrain (*Rosen* or *Rotlauff*). Writing about scurvy, which he termed Schurbauch and which would be standardized as *Skorbut* in the seventeenth century, Weyer lingered on the lesions it created.^74^ He then acknowledged that although similar terms and similar symptoms were found in the works of ancient authors, particularly Hippocrates, no author from antiquity adequately captured the distress, pain, intensity, and ubiquity of the symptoms of scurvy. For this reason, he wrote, "we may not term it *icteritiam nigram*" or any of the other classical possibilities, the only suitable term was the vernacular, the *Niedersächisch* term Schurbauch.^75^ In thinking about scurvy in terms of the physical appearance of symptoms in combination with the distress and pain of its physical presence, Weyer was consciously setting out a new standard for the identification of disease, one very much in line with the developments in the literature on [Other P-200] pox that preceded his treatise. Skin featured as the grounds for thinking about new disease more broadly.^76^

Finally, one kind of source that has yet to be mentioned is treatises on cosmetics and pharmaceutical literature, which were numerous in the period.^77^ It is within such sources that skin as a site of treatment most clearly linked practitioners and patients, and for many people this branch of medicine was also the most connected to daily life.^78^ Over the course of the century new materia medica aimed at skin proliferated, while new ingredients for pharmaceutical consideration took skin as their focal points. In Italy this can be seen clearly in the proliferation of books of secrets, many of which counted numerous "new" recipes for skin, as for example recipes for whitening the skin, easing wrinkles, or smoothing out hands in *I secreti de la signora Isabella Cortese*.^79^ In Germany, possibly due to the popularity of the publication of Hieronymus Brunschwig's *Liber de arte distillendi* in 1496, even translations of books marketed elsewhere as cosmetics, or secrets, were mostly sold as pharmacy, composing the genre commonly known as *Artzneybücher*.^80^ Such books mirrored the increasing presence of skin in other medical texts, a process illustrated by the 1568 *Artzneybuch* by Christoph Wirsung. Wirsung was a university-educated physician, but *Artzneybuch* was written in the vernacular.^81^ Some nine hundred pages long, *Artzneybuch* organized its contents head to foot, in the classic Galenic mode. However, alongside its sections on the head, the limbs, the torso, and so on, it included a whole section on skin. Wirsung titled the section "Diseases of the Whole Body" since a disease on skin could, [Other P-201] at least in theory, spread everywhere. (Wirsung also described skin as the "clothing of the body," a phrase Jessen would repeat more than twenty years later.) Twenty-one chapters followed, each broken into sections distinguishing the great variety of infirmities within the disease category. The correlation between "diseases of the whole body" and "skin" was clear enough that the first English translation of Wirsung's text retitled the section "Skin Diseases."^82^ Speaking to lay consumers and domestic practitioners, Wirsung's text provides yet more evidence that a general acceptance of skin disease formed part of the marketplace of medical ideas.

To summarize, what we see within the literature on the pox is a number of developments that happened in other fields as well—namely, a growing concentration in print on the visual signs provided by skin as well as an emerging consensus among medical practitioners on the role of skin in defining at least one important disease and the use of skin as a material to diagnose and ultimately treat it as well. Jon Arrizabalaga, John Henderson and Roger French have shown how pox was gradually accepted as a disease category, and Claudia Stein, while cautioning against overattributing medical change to pox, has affirmed the importance of pox as a phenomenon in sixteenth-century medical practices of diagnosis and treatment.^83^ Importantly, the position of pox was always essentially conceived of in relation to other skin diseases. Physicians and barber-surgeons alike contributed to the printed discourse on pox, and one thing that considering this literature brings to the fore is the degree to which techniques and procedures gleaned from surgery informed and indeed were increasingly coming to define skin.

In his writing about skin, Jessen was making a learned intervention into the longer movement of the subject of the Holy Roman Empire, which, as we have touched on already, began (textually) with vernacular experts and only latterly appeared in Latin. As his status suggests, surgery would not retain ownership of skin. As physicians became more interested in disease, they gradually co-opted surgery and also skin. In this respect, Jessen is a perfect foil. We have seen how skin emerged from a new focus on practice, evident in texts not otherwise overtly concerned with skin. It is time to see how a text about skin, which might otherwise be dismissed as an abstruse academic disputation, in fact engaged with similar themes of practice.[Other P-202]

## Jessen: Skin, Surgery, and Procedure

The year of *De cute*'s publication, 1601, was significant for Jessen. In 1597, he had become rector of Wittenberg, where he had already succeeded in combining appointments to three chairs, surgery (his original position, in 1594), anatomy, and botany.^84^ Throughout his time at Wittenberg, Jessen actively worked to expand his network, particularly in the direction of the imperial court of Rudolf II. Following a visit by Tycho Brahe to Wittenberg, Jessen began correspondence with the Danish astronomer and, through him, with Johannes Kepler.^85^ In 1600, Jessen visited Brahe's castle in Benátky as well as the astronomer's residence in Prague. It was during this visit that Jessen was responsible for carrying out the first public dissection outdoors in Prague, where, he later boasted, thousands of people gathered to watch him.^86^ Jessen quickly wrote up and published an account of this dissection, *The Prague Anatomy*, which appeared in 1601, dedicated to Emperor Rudolf II.^87^ The same year he published his most important work, the *Institutiones chirurgices* (The Surgical Lessons).^88^ The appearance of *On Skin*, which was also published in 1601, thus coincided with his most important medical contributions and should be seen in this vein. In contextualizing the emergence of *On Skin*, Jessen's other 1601 texts reveal the idiosyncrasies of their author's take on medical knowledge. They show how he married his exposure to Italian and German medicine and how he managed his career while developing his medical interests. Jessen relied on anatomy as a means of self-promotion, while folding it into an expanded remit for surgery and an intellectual interest in disease and treatment. Perhaps most significantly, Jessen used editorial strategies of organizing text and metadata as a framework to think through new ways of practicing medicine.

For example, *The Prague Anatomy* functioned almost exclusively as a means of self-advertisement. It did this most obviously by revolving around [Other P-203] an event, publicizing the allegedly popular and novel dissection carried out by Jessen a few months earlier. As a text Jessen's work closely follows Vesalius, although the text is small and cheap, containing none of the interventions designed to make it readable (illustrations, tables of contents, diagrams, etc.). Its cramped and cheap format almost masks the changes it did make—a small number of amendments to the anatomy of the eye. These reflect Jessen's training with Fabrici and would go on to be farther drawn out by Kepler. Rather than investigate or interrogate anatomical knowledge, then, *The Prague Anatomy* more clearly served as a statement that Jessen possessed such knowledge. In important respects, this statement was successful (he moved to the imperial court a year later, in 1602) and testifies to the increasing value of anatomy to a professional medical career. At the same time, while this was a statement of anatomical knowledge, read next to his other works, it is equally clear that in the context of his larger medical career, dissection was subservient to disease and its surgical treatment.

In terms of both intellectual innovation and material features, Jessen's *Institutiones chirurgices* was a more significant text. This is in large part because it spoke to his practical interests. Omitting the statement of anatomical know-how with which artisanal surgeons often began, the text started with a summary of the work of surgery: "cutting the surface, joining the parts together, elimination of the superfluous parts and restoration of the lost parts."^89^ While talk of the continuity and dissolution of surface guided traditional surgical works from Guy de Chauliac through Hans von Gersdorff, Jessen's inclusion of the "restoration of the lost parts" in particular spoke to an expanded remit for surgery, whose confidence lay in new methods and procedures developed in the sixteenth century.^90^ In general, when it came to surgery, Jessen emphasized rather than avoided the manual nature of the art. The body of his text is absent of the kinds of lengthy discussion of Galen or Hippocrates that characterizes the writing of his contemporaries, such as Mercurialis. Jessen demonstrated great familiarity with a broad range of techniques and procedures, from treating mundane mouth ulcers to hair removal and clearing the urinary tract. He also lingered on subjects more traditionally the preserve of barber-surgery, appending a long treatise on the nature of bones and describing the matter of surgery as "skin, flesh and bones."^91^ Some of Jessen's procedures would enter the surgical mainstream, and others would [Other P-204] be promptly forgotten. For example, the 1624 collection of observations by Gregor Horst recounted the case of one Johannes Meier, whose penis had been so desperately injured that urine was escaping in an opening between the glans and the foreskin. He had been treated according to the procedure laid out in the *Institutiones chirurgices* and was surviving (though, Horst recorded, he remained in abject misery).^92^

Like *The Prague Anatomy*, *Institutiones chirurgices* was also dedicated to the Emperor Rudolf II, and even more so than the former text, Jessen harnessed his network to produce a densely credentialed work. Melchior Jöstel, Daniel Sennert, and Adam Theodor Siber all contributed epigraphs. Jessen's proemia makes a very clear statement about the legacy within which one should read his writing on skin, referencing, among others, Tagliacozzi, Montagnana, Della Croce, and Fabrici as inspirations for the text. This summoning of contemporary expertise is important because it positions the text within a spectrum of practice linking surgery, anatomy, and a new focus on curative treatment of disease. It is thus easy to overlook that in fact Jessen *was* doing something new.

With surgical practice in mind, the most interesting novelty of the book can be said to lie in its organizational structure, which conforms to neither of the two main models for surgical (or indeed medical) texts in the sixteenth century. For the most part surgical manuals, such as those by Ambroise Paré and Von Gersdorff, follow the parts of the body and the illnesses or ailments common to them. This was also the case with the developing genre of observationes. Although they are more commonly noted for their new modes of narrating and recording case studies and for the way in which they privileged empirical observation (or the physician as agent), observationes tended to unfold by the Galenic logic of the head to toe. In contrast to both these mainstream genres, Jessen's *Institutiones chirurgices* progresses by procedure rather than by the affected body part. So the first section includes two chapters: *de ustione* (cauterization), followed by *sectione* (amputation), while the second section progresses through setting broken bones and aligning fractures. Each of these chapters on method can involve quite different individual procedures, as for example amputation, which features the notorious episode of the erupted penis, but which basic method is also used for a wide range of more minor problems, from abscesses of all kinds to pestilential tumors.^93^ While this emphasis on procedure was quite novel, it was directly reminiscent of the emphasis on material, body, and technique within vernacular works on [Other P-205] pox and surgery mentioned in the section above. Like Franz Renner's emphasis on "materey," the categorization of procedure prioritized above all else the matter of the body, terms in which Jessen would go on to frame skin in new ways.

Jessen's preceding 1601 texts illustrate how he used organizational structures of his text to make medical change. His work thus brings together different approaches, using textual reorganization to slide together fundamental reorientations about the body, surgery, and the medical approach to both, while at the same time making steadfast claims to professional primacy, tied clearly to practice. This would be evident in his work on skin as well. But it is important not to overstate the novelty of his work. When he published his twenty-one theses, Jessen did so under the assumption that they would reflect general interest in skin rather than provoke it. We can think of Jessen as a publicist of skin then, situating Wittenberg (and subsequently Bohemia) in a larger European set of developments that were not about the dissemination of Italian humanist ideas but about knowledge being made concomitantly, in very different places, by men (and women) with diverse interests, often outside the best established medical structures or disciplines.

## "On Skin and the Conditions of Skin"

**Figure 5:**
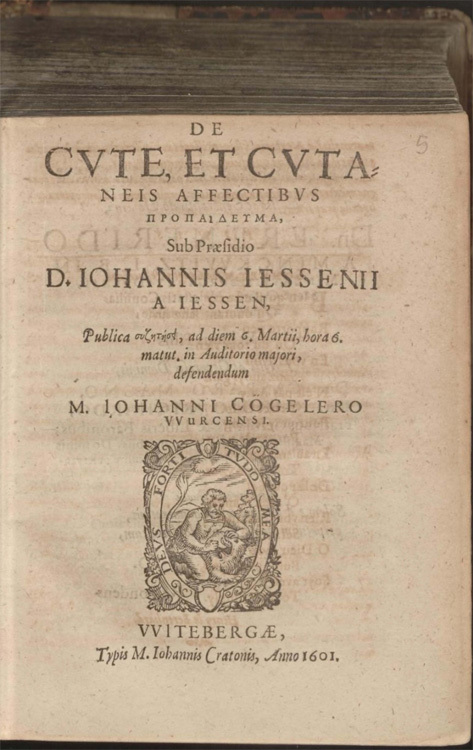
Jan Jessen's *De cute*, *et cutaneis affectibus* (1601).

It is within this context of a focus on organization, a peripheral but vigorous attention to changing surgery and anatomy, and a keen eye to self-promotion, that Jessen's text *On Skin* should be viewed. So what did this uncontroversial, concise text look like? It was unbound, twelve pages long, typeset in Wittenberg Garamond on low-grade paper by the university printer Johann Crato; today at least three copies survive (Figure 5).^94^ Like many other seventeenth-century university disputations, *On Skin* contained numbered theses, in this case twenty-one. These moved forward with some overlap, only brief elaboration, and varying degrees of evidence. It should by now be clear that *On Skin* was no breakthrough text, to be characterized by great novelty or innovation. Rather, in terms of content, Jessen's text is distinguished by its clarity of focus, by its unequivocal position on the existence of skin disease, by the language of matter in which he analyzed it, and, perhaps most notably, by his ability to marry the impulses of very different and sometimes downright conflicting medical schools of thought. Here, Jessen's magpie-like intellect came fully to life.[Other P-206]

[Other P-207]

Jessen's first thesis lays out the foundation for studying skin in terms of its relationship to the body. "To begin to look at this net from the outside, by the grace of God, you will be able to ascertain specific knowledge of the conditions of the whole human body and its arrangements, to understand and cure illnesses, not only those that are inside the body,—made evident by evils—but because of those [signs] outside too."^95^

Like his mentor Fabrici, Jessen's anatomical assumption here was that study of one part of the body (in this case skin) would allow for knowledge of the whole body. But Jessen goes further than Fabrici since he situated this insight not simply in the context of knowledge of the perfect body (i.e., anatomy), but knowledge of the diseased body (i.e., medicine). The same thesis stated a clear bodily purpose for skin, since through external faults of the body "not only was one or another part of the body or [its] workings interrupted, at the same time the beauty and the splendor of the body was ruined and disfigured."^96^ Although brief, this was an argument that Jessen would elaborate in several further theses. It laid out the grounds for the existence of skin disease, drawing together the different threads of medical interests, treatments, and demands that had given rise to skin in other literatures—namely, the skin as a barrier to the body, providing its defense, and the porous nature of skin, maintaining the integrity of the body's emissions, temperature, and physiognomic integrity (or beauty). Such ideas were not intended to contradict Galen (indeed, thesis II attributed them to his authority) but, as should by now be clear, nor were they straightforwardly orthodox. Jessen was influenced by contemporary writers, and much like his allegiance to Fabrici, he was untroubled by assimilating ideas from very different strains of thought. Thesis VIII for example breezily states that Galen and the ancients distinguished cutis from cuticula "and I am not disposed to refute them," while only a sentence later he does so, by describing a third element to the skin after Piccolomini: "For Piccolomini there are more classes of skin . . . the link: the skin is linked tightly to the forehead, to the hands, to the feet, and also to the lips, although with the remaining parts of the body, in truth, it sticks only more loosely."^97^ The turn to a natural philosopher, rather [Other P-208] than a medical writer, suggests not only widespread interest in skin and the eclecticism of Jessen's sources of authority but also the thoroughness with which he viewed the material qualities of skin, which were not just its practical accidents but essential to it.

Having described the anatomical nature of skin, Jessen moved on to his key concern—the presence and nature of diseases of the skin. Thesis IV observes the ubiquity of skin disease. "By its situation nevertheless the skin is liable to many accidents."^98^ Such language was reminiscent of surgery. Jessen's categorization of accidents caused by external forces or internal disruption mirrored traditional surgical ideas about wounds, which were described as dissolutions of the continuity of the surface, either by external or internal causes. Thesis XII clarifies, "Skin conditions are intemperate things that spoil the moderation of the skin."^99^ According to Jessen, this moderation could be disturbed in one of two ways. Skin could increase, as in the case of protuberances, warts, and pustules, or skin could diminish, its continuity destroyed by fissures, as in the case of impetigo or ringworm. Thesis XVII described skin ailments as "faults" that "can be touched" and had either "roughness or hardness," or faults of ugliness, defined by color and blemish.^100^ Either kind of skin disease could be odiferous or have stench, an idea that was of great important to Mercurialis before him. In essence then, as Jessen concluded by summarizing, disease struck skin in one of two ways. Either it attacked the vital function provided by the porosity of skin—a disease according to practice—or it attacked the attractiveness of skin, which provided safety to skin from all living things.

The significance of Jessen's summary here lay less in its substance and more in his willingness to articulate it in such a format. Much of what Jessen said can be found at greater length in Mercurialis or Giovanni Tomasso Minadoi, but his brevity, format, and structure formed a literal point of departure from the literary aims of the Italian writers. Comparing it explicitly with Mercurialis's text reveals this. As already mentioned, *De morbo cutaneis* followed the classical progression from head to toe. The first book featured diseases of the skin of the head, the second, diseases of the skin of the body. The whole volume was accompanied by a treatise on excretions, in which book 1 focused on urine, book 2 on excrement, [Other P-209] and book 3 on sweat, tears, spit, mucus, and earwax. There were numerous points of contact and overlap with Jessen. The question of texture was imperative to Mercurialis as well—the categorization of roughness and smoothness ran throughout his disease classification.^101^ The most compelling difference that reading these texts together produces is the clarity around disease categories provoked by Jessen's emphasis on surgery as well as the relative inattention Jessen paid to reconciling his findings with classical authorities. For Mercurialis, the question of diseases of the skin was to be considered alongside diseases of other parts of the body. Skin thus appeared as an organizational category within a text itself organized in traditional fashion. By contrast, Jessen's theses focused specifically on the question of whether to call such faults diseases of the skin. Skin disease was here not merely a window onto a set of diseases but an important question in and of itself. Jessen's text reveals a core structure around the ideas of skin that were derived not from Galen but from surgical practice.

We can see the implications of this play out in the way that he treated skin as a material substance. In the third thesis of his text, Jessen laid out the grounds for studying skin. "Finally," he wrote, "it is the clothing (*tegument*) of the entire body . . . for Plato a great net, for Hippocrates a chain of all the parts of the body, for it is the sheath of all the organs of the body. Namely a fortification and almost a bulwark, it evades by thickness and density."^102^ Skin as clothing, as a net, as a chain, as a sheath, as a fortification, or a bulwark—in this list, Jessen was in one way pointing out the multiplicity of previous treatments of skin, but he was also making unequivocal its material nature, embedding a concept of the body in its attributes, characteristics, and material properties. This continued to guide his work throughout the text. Thesis VI addressed the accidental qualities of skin: color, texture, feel. Thesis VII stated that it was necessary for skin to be temperate. Thesis VIII—particularly interesting—stated, "The skin is linked tightly to the forehead, to the hands, to the feet, likewise to the lips: In truth with the remaining parts of the body it sticks only more loosely."^103^ (There is something deeply fascinating about the [Other P-210] idea of a physician and anatomist pondering the way in which skin sticks to the body.) The preoccupation with the problematic nature of skin as a material that did not possess a shape continued in thesis IX: "The skin is perforated, so that it resembles a sieve (*cribrum*) with innumerable passages, which are obvious to the sense. . . . The shape is not particular to the skin, but copies the entire body. . . . The surface is not rough, nor very thick, nor hard, nor very damp, but has everything in moderation."^104^

Like Von Gersdorff and other Renaissance medical writers, in attempting to summarize, define, and describe skin, Jessen turned to metaphor and analogy. But unlike Von Gersdorff, the metaphor here rebounded to tell the reader something about the body.

From a literary standpoint, in Jessen's text, skin pinpoints moments of empirical observation, but also their limits—the way in which, when forced to provide explanation for how the body works, Jessen turns away from what he has seen and veers into the realm of imagination. This went beyond linguistic command or linguistic affectation. It had implications for the way in which skin, as material, mattered to the body's senses and to the relationship between body and sense.

In his 1601 *Prague Anatomy*, Jessen described skin as the organ of touch. This was a commonplace rejection of Aristotle, who argued that flesh was the organ of touch. Andreas Vesalius, Ambrose Pare, Volcher Coiter, Hieronymus Mercurialis, and Giulio Cesare Casseri, among many others, all explicitly rejected Aristotle and stated that skin was the organ of touch. But while thinking of skin as the organ of touch was an important recategorization of skin (and touch), until new attention began to be paid to skin and disease, there was little evidence that this change affected medical categories or conceptions of disease.^105^

For Jessen, however, skin as material and skin as sense were related to disease. Not only could disease be detected by the material qualities of skin vis-à-vis touch (its roughness, smoothness, and hardness), smell (its stench), and sight (its fissures and growths), but disease could be evident in the corruption of the relationship between skin and sense as well. This is particularly evident in thesis XIII. Sandwiched between consideration of warts and consideration of hairs, nails, and other skin-adjacent body parts, the thesis deals with differences between lesions, which, following his general distinction between modes of disease, Jessen divides in two: [Other P-211] "those that are partly natural and those that are partly animal." The natural "are those same lesions that are conditions that undermine the structure. Whereas the animal ones are faults that can be chiefly perceived by the sense, that is all kinds of itchiness and all itchy conditions."^106^

Itchiness here conforms to neither of the two categories of disease previously established by Jessen, that is, diseases caused by external forces or by imbalance of internal forces. In this text, itchiness (*pruritus* is the term used by Jessen, as indeed it is used by physicians today) is a sensation. Pruritus appeared in other texts, including most notably Mercurialis, who devoted a lengthy chapter to it in book 2 of *De morbis cutaneis*. Mercurialis classified it as a disease of the smooth skin, since no swelling, ulceration, or excoriation appeared. Pruritus could be mild (relieved by itching) and severe (requiring alleviation by other means)—and when sensation occurred, Mercurialis termed it a form of pain "dolor" and hypothesized that physiologically it was due to some dissolution of continuity within the skin's multiple layers, or due to thin humors, trapped within, a damaged sense of touch.^107^ Mercurialis contrasted the pleasure of relief from itchiness with the pain it causes. Jessen, however, placed the sensation of pruritus apart from the pain/pleasure dichotomy. Partly animal, and perceived chiefly by the senses, this is very different configuration than that of Mercurialis—and it is telling that classical figures such as Aristotle appear nowhere in this section of the text.

It was only with Samuel Haffenreffer's 1660 text, the *Nosodochium*, that pruritus or itchiness would it be formally afforded the status of a disease of the senses or a dermatological category.^108^ But in Jessen we can already see a clear link between pruritus and the senses, one that is not present in other sixteenth-century works on skin. Itchiness may not seem like a significant medical discovery, but the grounding of physical sensation in medicine was one of the most important changes of the seventeenth century. Itchiness, of all things, therefore draws together the variety of things we see as determining the emergence of skin in the sixteenth century—surgery, empirical observation, anatomical knowledge, and ideas of touch and tactility.[Other P-212]

## Conclusion

The story of skin is not one of new ideas. The basic Galenic conceptions of the body were not new in the sixteenth century, and their substance did not effectively change. The texts that were written about skin were not "origin stories." They produced new thematizations of skin, but they were not about nor did they cause the birth of dermatology. This is evident in Jessen's text, which should be seen as a capstone, rather than a moment of great change or progress. And yet, as *On Skin* also shows, there were significant developments in the way in which skin was conceptualized and treated in learned medicine, particularly in regard to its status vis-à-vis disease.

The presence and consideration of skin disease speak directly to the intricate tension facilitated by Galenic medicine, the "pliancy" as Mary Lindemann has termed it, that allowed Galenic categories to encompass distinctly un-Galenic subcategories.^109^ Historians who have written about skin in the sixteenth century have assumed that because skin disease was not explicitly Galenic it could not exist.^110^ Historians who have written about skin in the seventeenth and eighteenth centuries have treated it with far more sensitivity.^111^ But while they have emphasized its ubiquity and its role in facilitating common cultures of semiotics, they have nonetheless assumed this, in some senses, to be a visible marker of the decline of Galenism. Nuancing this by seeing skin within the framework of Galenic medicine allows us to more fully appreciate not only the complexity of Galenic theory but also the more meaningful complexity of its practice, the way in which its learned critics shored up orthodoxy, all the while continually folding into it a great heterodoxy of lay and artisanal practices. The emergence of skin as a medical subject and the way it facilitated and shaped developments in concepts and categories of disease provide critical insight into what happens when a framework remains stable but its materials fundamentally change.

Like Jan Jessen's work, this is not a new observation. Nancy Siraisi, Ian Maclean, and many others have all drawn attention to the way in which textual forms inherited from scholasticism or convention could contain, facilitate, and subvert new forms of information, new scholarly arguments, [Other P-213] and new modes of thought.^112^ The reconfiguration of learning they have uncovered and its changing relationship to practice are key to the way we now think about the medical Renaissance. But text was only one part of this. Underneath the textual veneer of Galenism, which linked physicians, surgeons, apothecaries, patients, and a broad variety of practices, the matter of medicine was rapidly changing. Skin was one thread in this broader change. The influx of New World ingredients, the growing number of plants, broadening familiarity with more complex methods of preparation, including distillation and decoction, and tools and surgical techniques, the dissemination of medical knowledge in the form of books, and the changing organization of that knowledge through different genres of books all helped reshape the stuff of medical practice, while retaining the lines of medical authority.

As Jessen's text makes clear, sixteenth-century thinkers themselves were quick to pick up on the rich possibilities of skin and use it—to promote themselves, to conceptualize disease, to define practices, and ultimately to develop treatments. According to Jessen, knowledge about skin was not just a feature of medical practice, it was a medical material, one that encompassed multiple models for the body, which could and did coexist (nets, bulwarks, fortresses, clothing, etc.). The minor but meaningful contributions Jessen made to the development of medical knowledge around skin drew on surgery and anatomy, on empirical observation, but equally on a rich vein of metaphor and a healthy dose of professional ambition. This is evident in the way that skin functioned as a site of change and a vital surface for sense and sensation. As a conclusion to this article, I suggest that the advances actually made were less important than the way in which such advances were made. Skin offered a different way to think about the sixteenth-century body, one defined less by humors and complexions and more by procedures, treatments, and disease. For Jessen, as for others of his ilk, skin sat not just at the juncture of medical conceptions of matter and sense but at the very border of the body and its medical possibilities.[Other P-214]

## Footnotes

1
PickFriedel, "Joh. Jessenius De Magna Jessen. Arzt und Rektor in Wittenberg und Prag Hingerichtet am 21. Juni 1621," Studien zur Geschichte der Medizin 15 (1926): 1–315, here 95–96. On disputations, see FriedenthalMeelis, "Nominal Definition in the Seventeenth-Century University Disputations of the German Cultural Space," Hist. Universities 29 (2016): 65–87; FreedmanJoseph S., "'Professionalization' and 'Confessionalization': The Place of Physics, Philosophy, and Arts Instruction at Central European Academic Institutions during the Reformation Era," Early Sci. Med. 6 (2001): 334–52.

2
JessenJan, De cute, et cutaneis affectibus propaideuma (Wittenberg: Johann Crato, 1601).

3 Ibid., thesis XIX: "Verum controversia est, dicendanè morbis cutis vitia. . . ."

4
Of course concepts of disease are historically and socially constructed, and no one exhaustive definition prevailed. See StolbergMichael, Experiencing Illness and the Sick Body in Early Modern Europe (London: Palgrave Macmillan, 2011); RosenbergCharles E., "Disease in History: Frames and Framers," Milbank Quart. 67 (1989): 1–15; HaysJ. N., The Burdens of Disease: Epidemics and Human Response in Western History (New Brunswick, N.J.: Rutgers University Press, 2009).

5
Ideas that diseases could be external "entities" were also in circulation, although it was widely thought that they entered the body due to an underlying humoral imbalance. WearAndrew, Knowledge and Practice in English Medicine, 1550–1680 (Cambridge: Cambridge University Press, 2009); LindemannMary, Medicine and Society in Early Modern Europe (Cambridge: Cambridge University Press, 2010); SiraisiNancy, Medieval and Early Renaissance Medicine: An Introduction to Knowledge and Practice (Chicago: University of Chicago Press, 1990).

6
Galen, Method of Medicine, trans. JohnstonIan (Cambridge, Mass.: Loeb, 2011), 180–81.

7
MercurialeGirolamo, De morbis cutaneis (Venice: Paul Aicardi, 1572).

8
Stolberg, Experiencing Illness (n. 4), 107.

9
Wear, Knowledge and Practice in English Medicine (n. 5), 106.

10
HennepeMienekete, "Of the Fisherman's Net and Skin Pores. Reframing Conceptions of the Skin in Medicine, 1572–1714," in Blood, Sweat, and Tears—The Changing Concepts of Physiology from Antiquity into Early Modern Europe, ed. HorstmanshoffManfredKingHelenZittelClaus (Leiden: Brill, 2012), 522–48.

11
The most comprehensive source for Jessen's biography remains the early twentieth-century study by Pick, "Joh. Jessenius" (n. 1). On Jessen and the revolt, see SousedíkStanislav, "Jan Jesensky as the Ideologist of the Bohemian Estates' Revolt," Acta Comeniana 11 (1995): 13–24; *Eigentliche Abbildung dess Process der Pragerischen Execution* (1621); *Kurze, jedoch gantz gewisse und warhafftige Relation mit was Ordnung die Böhmische herzen/als Graffen/ Herren/ Rittern und Burger stands Personen zu Prag/ den 21 Junii/dieses 1621 Jahrs/justificirt und hingerichtet worden* (1630).

12
Variously described as Bohemian, Polish, and German, Jessen in fact styled himself as a Hungarian knight. See TeszelskyKees, "The Hungarian Roots of a Bohemian Humanist: Johann Jessenius a Jessen and Early Modern National Identity," in Whose Love of Which Country? Composite States, National Histories and Patriotic Discourses in Early Modern East Central Europe, ed. TrencsenyiB.ZászkaliczkyM. (Leiden: Brill, 2010), 315–32.

13
LindemanMary, *Health and Healing in Eighteenth-Century Germany* (Baltimore: Johns Hopkins University Press, 1996); SteinClaudia, *Negotiating the French Pox in Early Modern Germany* (Burlington, Vt.: Ashgate, 2009); HammondMitchell, "Paracelsus and the Boundaries of Medicine in Early Modern Augsburg," in Paracelsian Moments: Science, Medicine and Astrology in Early Modern Europe, ed. WilliamsGerhild ScholzGunnoeCharles D. (Kirksville, Mo.: Truman State University Press, 2002), 19–34; StürzbecherManfred, "The Physici in German-Speaking Countries from the Middle-Age to the Enlightenment," in The Town and State Physician in Europe from the Middle Ages to the Enlightenment, ed. RussellAndrew (Wolfenbüttel: Herzog August Bibliothek, 1981), 123–31.

14
MurphyHannah, A New Order of Medicine: The Rise of Physicians in Reformation Nuremberg (Pittsburgh: University of Pittsburgh Press, 2019); MendelsohnJ. AndrewKinzelbachAnnemarieSchillingRuth, eds., Civic Medicine: Physician, Polity, and Pen in Early Modern Europe (London: Routledge, 2019).

15 Jan Jessen, *De animae humanae immortalitate dissertatio* (Leipzig, 1587). See also Pick, "Joh. Jessenius" (n. 1), 294.

16
KlestinecCynthia, "Renaissance Surgeons: Anatomy, Manual Skill and the Visual Arts," in Early Modern Medicine and Natural Philosophy, ed. DistelzweigPeterGoldbertPeter BanjaminRaglandEvan R. (Dordrecht: Springer, 2016), 43–58; SavoiaPaolo, "Skills, Knowledge and Status: The Career of an Early Modern Italian Surgeon," Bull. Hist. Med. 93 (2019): 27–54; PalmerRichard, "Physicians and Surgeons in Sixteenth-Century Venice," Med. Hist. 23 (1979): 451–60; GrellOle PeterCunninghamAndrewArrizabalagaJon, eds., Centres of Medical Excellence? Medical Travel and Education in Europe 1500–1789 (Aldershot: Ashgate, 2010).

17
KlestinecCynthia, Theaters of Anatomy: Students, Teachers and Traditions of Dissection in Renaissance Venice (Baltimore: Johns Hopkins University Press, 2011).

18 Ibid., 55–56.

19 On this complex interplay, see Savoia, "Skills, Knowledge and Status" (n. 16).

20
SavoiaPaolo, Gaspare Tagliacozzi and Early Modern Surgery: Faces, Men and Pain (London: Routledge, 2019); BondioMariacarla Gadebusch, Medizinische Ästhetik: Kosmetik und plastische Chirurgie zwischen Antike und früher Neuzeit (Munich: Fink, 2005).

21
AnzieuDidier, *The Skin Ego*, trans. TurnerChris (New Haven, Conn.: Yale University Press, 1989); ConnorSteven, The Book of Skin (Ithaca, N.Y.: Cornell University Press, 2003); MaurettePablo, The Forgotten Sense: Meditations on Touch (Chicago: University of Chicago Press, 2018); BenthienClaudia, Skin: On the Cultural Border between Self and World, trans. DunlapThomas (New York: Columbia University Press, 2002).

22
FendMechthild, Fleshing Out Surfaces: Skin in French Art and Medicine, 1650–1850 (Manchester: Manchester University Press, 2016); BohdeDaniela, "Skin and the Search for the Interior: The Representation of Flaying in the Art and Anatomy of the Cinquecento," in Bodily Extremities: Preoccupations with the Human Body in Early Modern European Culture, ed. EgmondFlorikeZwijnenbergRobert (Ashgate: Aldershot, 2003), 10–47. See also OlsonTodd, "Ribera, Bartholomew and the Empire of Resemblances," Oxford Art J. 41 (2018): 171–95.

23
ReinarzJonathanSienaKevin, eds., A Medical History of Skin: Scratching the Surface (London: Pickering and Chatto, 2013). Reinarz and Siena's seminal volume has done much to build up the picture of skin since the eighteenth century, but as they clearly state in their introduction, "The first suggestion for future work must entail a companion volume on the medieval and early modern periods" (3).

24
Galen, Hygiene, I. Books 1–4, trans. LoebIan Johnson (Cambridge, Mass.: Harvard University Press, 2018), 250–51; ZieglerJoseph, "Skin and Character in Medieval and Early Renaissance Physiognomy," Peau humaine/pelle umana, Micrologus 13 (2005): 511–53. See also SteudelJohannes, "Bau und Funktion der Haut in der Antike," Studium Generale 10 (1964): 583–88.

25 This is in keeping with Helen King's observation that "while anatomical structures can be discovered by dissection, function can less readily be seen." Helen King, "Introduction," in Horstmansoff, King, and Zittel, *Blood, Sweat, and Tears* (n. 10), 1–24, quotation on 10.

26
VesaliusAndreas, On the Fabric of the Human Body: Book I. The Bones and Cartilages, trans. RichardsonWilliam Frank (San Francisco: Norman, 1998), 8.

27
PetcuElizabeth, "Amorphous Ornament: Wendel Dietterlin and the Dissection of Architecture," J. Soc. Architectural Hist. 77 (2018): 29–55, 29.

28
DudenBarbara, The Woman Beneath the Skin: A Doctor's Patients in Eighteenth-Century Germany, trans. DunlapThomas (Cambridge, Mass.: Harvard University Press, 1991).

29
Isidore of Seville, The Etymologies of Isidore of Seville, trans. BarneyStephen A. (Cambridge: Cambridge University Press, 2006).

30
Avicenna, Liber canonis totius medicinae, ed. ChampierSymphorien (London, 1522).

31 Celsus's works appeared in print in 1478.

32
In 1986, the physician Richard Sutton published a translation of the first book of Mercurialis's text. Although the book is still useful, it should be noted that by his own admission, Sutton took many liberties with the original translation, which was in fact made by a Latin teacher, Irene Blase. SuttonRichard L., A Sixteenth Century Physician and His Methods: Mercurialis on Diseases of the Skin. The First Book on the Subject (1572) (Kansas City: Lowell Press, 1986).

33
Sutton, Sixteenth Century Physician (n. 32), 12.

34 Ibid., 13.

35
SiraisiNancy, "History, Antiquarianism and Medicine: The Case of Girolamo Mercuriale," J. Hist. Ideas 64 (2003): 231–51. See also AcrangeliAlessandroNuttonVivian, eds., Girolamo Mercuriale: Medicina e Cultura Nell'Europa del Cinquecento (Forlì: Leo S. Olschki, 2008).12852408

36
MercurialeGirolamo, De arte gymnastica libri sex (Venice: Junta, 1569).

37
MercurialeGirolamo, De decoratione (Venice: Meietus, 1585). Sutton erroneously states that the first edition was published in Frankfurt in 1578; in fact it was a second edition, published in 1587.

38
MercurialeGirolamo, De decoratione liber (Frankfurt: Johann Wechel, 1587). It is noteworthy that *De decoratione* had particular traction in Germany, where its second edition was printed in Frankfurt in 1587.

39
WeisserOlivia, "Boils, Bumps and Wheals: Reading Bumps on the Body in Early Modern England," Soc. Hist. Med. 22 (2009): 321–39.

40 Acrangeli and Nutton, *Girolamo Mercuriale* (n. 35); Te Hennepe, "Of the Fisherman's Net and Skin Pores" (n. 10). Using Mercurialis only as a starting-off point, Hennepe argues strongly for the importance of the microscope in changing early modern ideas about skin and pores.

41
HeinrichsErik A., Plague, Print and the Reformation: The German Reform of Healing, 1473–1573 (London: Routledge, 2017).

42
TelleJoachim, Pharmazie und der gemeine Mann. Hausarznei und Apotheke in deutschen Schriften der frühen Neuzeit (Wolfenbüttel: Herzog August Bibliothek, 1982).

43
PomataGianna, "Sharing Cases: The Observationes in Early Modern Europe," Early Sci. Med. 15 (2010): 193–236.2069539410.1163/157338210x493932

44
HausseHeidi, "The Locksmith, the Surgeon, and the Mechanical Hand: Communicating Technical Knowledge in Early Modern Europe," Technol. Cult. 60 (2019): 34–64; TaapeTillmann, "Distilling Reliable Remedies: Hieronymus Brunschwig's Liber de arte distillandi (1500): Between Alchemical Learning and Craft Practice," Ambix 61 (2014): 236–56; RankinAlisha, "Empirics, Physicians and Wonder Drugs in Early Modern Germany: The Case of the Panacea Amwaldina," Early Sci. Med. 14 (2009): 680–710; Telle, *Pharmazie und der gemeine Mann* (n. 42).

45
GersdorffHans Von, Feldbuch der Wundartzney (Strasburg: Riel, 1517).

46
CaulhiacoGuigonis de (Guy de Chauliac), Inventarium sive chirurgia magna, vol. 1: *Text*, ed. McVaughMichael R. (Leiden: Brill, 1997) and vol. 2: Commentary, ed. McVaughMichael R.OgdenMargaret S., (Leiden: Brill, 1997).

47 Von Gersdorff, *Feldbuch der Artzney* (n. 45), i (in the margin): Cutis: Deshalb an der hut ansufahen ist, wann die bekumet an dem ersten unnd von uffzen als ein rynd des baums. Und ist ein deckel des leibs / uff den faede men der sennsen und aderen zusamen gesetzt / beschaff en die anderen glyd z beschirmen.

48 Hieronymus Brunschwig, *The noble experyence of the vertuous warke of surgeri* (1525), 6. The 1525 English translation erroneously attributes the text to Brunschwig, but as the illustrations suggest and the text makes clear, this is a translation of Von Gersdorff's text, not Brunschwig's.

49
CavalloSandra, Artisans of the Body in Early Modern Italy: Identity, Families and Masculinities (Manchester: Manchester University Press, 2007); SmithPamela, The Body of the Artisan: Art and Experience in the Scientific Revolution (Chicago: University of Chicago Press, 2004).

50
HerzMichael, Das Ackerwerk (Strasbourg: Riel, 1538); Savoia, Gaspare Tagliacozzi (n. 20); SavoiaPaolo, "Nature or Artifice? Grafting in Early Modern Surgery and Agronomy," J. Hist. Med. & Allied Sci. 72 (2017): 67–86.10.1093/jhmas/jrw03928168282

51 Kevin Siena and Jonathan Reinarz, "Scratching the Surface," in Reinarz and Siena, *Medical History of Skin* (n. 23); Weisser, "Boils, Bumps and Wheals" (n. 39).

52
Stein, Negotiating the French Pox (n. 13); BlochIwan, Der Ursprung der Syphilis: eine medizinische und kulturgeshichtliche Untersuchung (Jena: Fischer, 1901–11). Luke Demaitre shows a similar process at work in his important volume on leprosy. See Demaitre, Leprosy in Premodern Medicine: A Malady of the Whole Body (Baltimore: Johns Hopkins University Press, 2007).

53
UlseniusTheodoricus, "Vaticinium in epidemicam scabiem," in Theodorici Ulsenii Phrish Vaticinium in epidemicam scabie*m*, ed. FuchsC. H. (Göttingen: Verlag der Dieterichschen Buchhandlung, 1850).

54 Ibid., lines 1–2: "Nuper inauditam scabiem mutabile vulgus / Clamat et arbitrio doctae stipenda turbae"; lines 13–14: "Non ego retrogradus flecto giramine gressus / Roscida signifero quamquam soror evolat arcu."

55
ArrizabalagaJonHendersonJohnFrenchRoger, The Great Pox: The French Disease in Renaissance Europe (New Haven, Conn.: Yale University Press, 1997), 26.

56
SteinClaudia, Negotiating the French Pox (n. 13), 33.

57
HundtMagnus, Eyn kurtzes und sehr Nutzbarlichs Regiment wider die schwynde und erschreckliche Krankheit der Pestilentz (Leipzig: Schumann, 1529). On Hundt and anthropologia, see SantingCatrien, "Magnus Hundt's and Galeazzo Capra's Quest for Humanity," Hist. Anthrop. 29, no. 3 (2018): 1–29; RossTricia, "Anthropologia: An (Almost) Forgotten Early Modern History," J. Hist. Ideas 79 (2018): 1–22.

58
Hundt, Eyn kurtzes (n. 57), 3–4.

59
CavalloSandraStoreyTessa, Healthy Living in Late Renaissance Italy (Oxford: Oxford University Press, 2013).

60 Hundt, *Eyn kurtzes* (n. 57), esp. 8, 14. See also 19r–v, 20r–v.

61 Ibid., 13.

62 Ibid., 13. "Also auch feuchte coerper mit teglicher ubung und Regiment das sich neht zu truckheyet andern Welcher art dy artzney auch sein sal durch welche ihre trunkken eyegeschafft aller gifft widderstehet als seint savreapff Tormentille Bibennl Diptan herba tunice Scabiosa Ochsenstzunge alnthwruz Encian Zitwer kraen eugelein Mellisa betonica metter odder bolvus armenius und terra sigillata."

63
Ibid., 19r–v. See also RennerFranz, Ein neues wohlgegründetes, nützliches und heilsames Handbüchlein gemeiner Praktik aller innerlicher und äusserlicher Arznei, so wider die erschreckliche, abscheuliche Krankheit der Franzosen moegen gebracht werden (Nuremberg: Gabriel Hain, 1557), fol. VII; Stein, *Negotiating the Pox*, (n. 13) 41.

64
ShotwellR. Allen, "The Great Pox and the Surgeon's Role in the Sixteenth Century," J. Hist. Med. & Allied Sci. 72 (2017): 21–33.2816828510.1093/jhmas/jrw038

65
ArrizabalagaHendersonFrench, Great Pox (n. 55), 34–35.

66 Renner, *Ein neues wohlgegründetes Handbüchlein* (n. 63).

67 Ibid., n.p., fol. 26r.

68 Savoia, *Gaspare Tagliacozzi* (n. 20).

69 Renner, *Ein neues wohlgegründetes Handbüchlein* (n. 63), chap. 2, IIIr: "Jarlang was davon mit theilen / das aber solche kranckheyt bey uns erblich / wie im amfang erzelt / haben wir wold dergleichen gnugsame Exempel in andern kranckheyt bey uns erblich / Als da etliche personen mit. Blasenstein / ander mit dem Podagra, Chiragra, Sciatica geplagt und teglich gekrenckt werden."

70 Pomata, "Sharing Cases" (n. 43).

71
LuisiniLuigi, De morbo Gallico, omnia quae extant abud omnes medico cuiscunque nationis (Venice: Zilettus, 1566).31545783

72 Seventeenth-century works doubled down on the category of skin diseases and on the place of pox within that category. See Wolfgang Hoefer, *Disputatio Medica De Elpehantia Sive Lepra* (1635); Henrich Petraeus and Johann Mylius, *Disputatio Hermita Juxta ac Dogmatica, De Elephantiasi seu Lepra Arabum* (Marburg, 1613); Jean de Varanda, *Opera omnia theorica et practica, 2. Tractatus de elephantiasi sue lepra item de lue venera* (1620).

73
WeyerJohann, Medicarum observationum rararum liber unus (Basel: Oporinus, 1567); Weyer, Artzneybuch von etlichen biss anher unbekandten vnnd vnbeschriebenen Kranckheiten (Frankfurt: Nicholas Basse, 1583).

74
MayerMaximilian, "Verständnis und Darstellung des Skorbuts im 17. Jahrhundert" (M.D. thesis, Julius-Maximilians-Universität Würzburg, 2012), 10–14.

75 Weyer, *Artzneybuch* (n. 73): "Denn im Schurbauch sehen wir / wie abscheuhlich das Zanfleisch mut blutigem Eyterfaulet / das Maul eytert und stinckt / die Schenckel und Beyn mit hesslichen Flecken und Mackeln geschendet / auch mit unheylbaren Schaeden geoeffnet / die Glieder verlahmet und verkrumet / unnd endtlcih die gantze gestalt des Leibs so erbarmlich geandert und verstellt wir / das man diese Kranckheit nicht icteritiam nigram oder lienes magnos Hippocratis, oder tertiam voluusis speciem, oder mit einigem andern ohn allein mit dem innlandischen Niderteutschen Namen zu recht nennen kan."

76
This continued, often with the same diseases. See GentilcoreDavid, "'Italic Scurvy,' 'Pellarina,' 'Pellagra': Medical Reactions to a New Disease in Italy, 1770–1815," in ReinarzSiena, Medical History of Skin (n. 23), 57–69.

77
See EamonWilliam, Science and the Secrets of Nature: Books of Secrets in Medieval and Early Modern Culture (Princeton, N.J.: Princeton University Press, 1994). See also introduction in LeongElaineRankinAlisha, eds., Secrets and Knowledge in Medicine and Science, 1500–1800 (Aldershot: Ashgate, 2012).

78
LeongElaine, Recipes and Everyday Knowledge: Medicine, Science and the Household in Early Modern England (Chicago: University of Chicago Press, 2018).

79
CorteseIsabella, I secreti de la Signora Isabella Cortese ne'quali si contengono cose minerali, medicinali, arteficiose & alchimiche & molte de l'arte profumatoria, appartenenti a ogni gran Signora. Con altri bellissimi secreti aggiunti (Venice, 1565). See also CampbellErin J., Old Women and Art in the Early Modern Italian Domestic Interior (Burlington, Vt.: Ashgate, 2015), 115–16.

80 See, for example, Hans Wecker's best-selling text that was a translation of Alessio of Piemontese. Johannes Jacob Wecker, *Kunstbuch* (1617).

81
WirsungChristoph, Ein new Artzney Buch Darinn fast alle eusserliche unnd innerliche Glieder dess menschlichen Leibs sampt iren Kranckheiten und Gebrechen (Heidelberg: Mayer, 1568); SchoferUlrikePfisterKathrin, Das Heidelberger Artzney-Buch 1568 des Christoph Wirsung: Naturheilkunde in der Frühen Neuzeit (Knittlingen: Bibliotheca-Palatina_Faksimilie-Verl, 2011).

82
WirsungChristof, The General practise of physicke Conteyning all inward and outward parts of the body (London: J. Legat, 1617), 551–621.

83
SteinClaudia, "The Meaning of Signs: Diagnosing the French Pox in Early Modern Augsburg," Bull. Hist. Med. 80 (2006): 617–48.1724254910.1353/bhm.2006.0142PMC2630158

84
HelmJürgen, "'Medicinam aspernari impietas est'—Zum Verhältnis von Reformation und akademischer Medizin in Wittenberg," Sudhoff's Archiv 83 (1999): 22–41.10475050

85
NejeschlebaTomáš, "The Relationship between Johannes Jessenius and Johannes Kepler," in Kepler's Heritage in the Space Age, Acta Historiae Rerum Naturalium Necnon Technicarum, vol. 10, ed. HadravováAlenaMahoneyTerence J.HadravaPetr (Prague: National Technical Museum, 2010), 136–42.

86
JessenJan, Anatomiae, Pragae anno 1600 abse se solenniter administratae historia (Wittenberg: Laurent Seuberlich, 1601). See also NejeschlebaTomáš, "Justification of Anatomical Practice in Jessenius's *Prague Anatomy*," Early Sci. Med. 21 (2016): 557–74.29727532

87 Jessen, *Anatomiae* (n. 86).

88
JessenJan, Institutiones Chirurgicae, quibus universa manu medendi ratio oftenditur (Wittenberg: Laurent Seuberlich, 1601).

89 Ibid., 2v.

90
MeliDomenico BertoloniKlestinecCynthia, "Renaissance Surgery between Learning and Craft," J. Hist. Med. & Allied Sci. 72 (2017): 1–5.2817315610.1093/jhmas/jrw043

91 Jessen, *Institutiones* (n. 88), 3r.

92
HorstGregor, Observationum medicinalium singularium libri quator (Ulm: Jonas Saur, 1625), 518; Jessen, *Institutiones* (n. 88), 39r.

93 Jessen, *Institutiones* (n. 88), 22r–25b.

94 Herzog August Bibliothek, Staatsbibliothek zu Berlin and Sächsiche Landesbibliothek.

95 Jessen, *De cute* (n. 2), thesis I: "Cum, adjuvante Deo, de humani corporis totius affectibus peculiari via differere, constitutum nobis sit, ab externis telam hanc ordiri visum est: non solum quod hi iis, quae intra corpus contingunt, malis evidentiores, sed quod externorum quoque morborum cognitio curatioque."

96 Ibid.: "Quod per externorum vitia non tantum unius quandoque vel alterius partis aut totius impediatur operatio, sed simul pulchritudo & decus corporis deturpetur dedecoreturq; Quo pacto nomine duplici tales existuntur molestiae."

97 Ibid., thesis VIII: "Quibus ego valde non repugno. Archangelo Picolominio tamen, plura cutis genera singenti."

98 Ibid., thesis IV: "Per suum tamen situm cutis multis obnoxia est offensionibus."

99 Ibid., thesis XII: "Cutanae affectiones vel spectant ad cutem, vel ad constitutionem cutis, vel quae hanc constituionem consequuntur. Sic temperamentum cutis vitiat intemperies quaeis."

100 Ibid., thesis XVII: "Tactilis qualitatus vitia sunt, asperitas cutis & callositas, veluti visibilis qualitatis turpitudo in colore consistit & maculis, non aliter ac odorabilis in foetoribus."

101
MercurialeGirolamo, De morbis cutaneis et omnibus corporis humani excrementis tractatus, (Venice: 1572); see esp. book 2, chap. 2.

102 Jessen, *De cute* (n. 2), thesis III: "Demum corporis totius tegumentum est, occasione sitis fuis, quoniam. 2. de usu part. 6. omnium caeterarum partium extima sit collocata. Platoni magna quadam nassa; Hippocrati omnium coporis partium vinculum, nobis omnium membrorum corporis vagina. Munimentum enim & quasi propugnaculum per crasitiem & denfitatem evadit."

103 Ibid., thesis VIII: "Nectitur cutis fronti, manibus, pedibus, item labiis, strictius: cum reliquis vero corporis partibus cohaeret paulo laxius."

104 Ibid., thesis IX: "Cutis, cribri instar, innumerabilibus spiramentis. . . . Superficies demum, nec aspera, nec levis nimis, nec dura, nec humid nimium, sed in mediocritate habet omnia. Sequitut continuitas."

105
HarveyElizabeth D., ed., Sensible Flesh: On Touch in Early Modern Europe (Philadelphia: University of Pennsylvania Press, 2002).

106 Jessen, *De Cute* (n. 2), thesis XIII: "Quae constitutionem cutis consequuntur in eo quod sit, illius operationes exsistunt, quae cum partim naturales, partim animales snt, sic etiam distinctas sustnet laesiones. Et naturalium quidem operationum laesiones easdam sunt affectiones, quae constitutionem ipsam labefactant. Animalium autem & potissimum sensitivarum vitia sunt pruritus omnes, omnesque, pruriginosae affectiones."

107 Mercuriale, *De morbis cutaneis*, 33v.

108
HaffenrefferSamuel, Nosodochium, In quo cutis eiqve adhaerentium partium, affectus omnes, singulari methodo, et cognoscendi et curandi fidelissime traduntur (Ulm: Kühn, 1660).

109
LindemannMary, Medicine and Society in Early Modern Europe (n. 5), 87.

110
Stolberg, Experiencing Illness (n. 4), 105–9.

111 Weisser, "Boils, Bumps and Wheals" (n. 39); Reinarz and Siena, *Medical History of Skin* (n. 23).

112
SiraisiNancy, History, Medicine and the Traditions of Renaissance Learning (Ann Arbor: University of Michigan Press, 2007); MacleanIan, Logic, Signs and Nature in the Renaissance (Cambridge: Cambridge University Press, 2007).

